# Management of infected bone defects of the femoral shaft by Masquelet technique: sequential internal fixation and nail with plate augmentation

**DOI:** 10.1186/s12891-024-07681-x

**Published:** 2024-07-17

**Authors:** Xiaoyong Yang, Xiaoyan Xu, Junyi Li, Muguo Song, Han Sun, Hu Zhang, Xijiao Zhang, Yongqing Xu, Jian Shi

**Affiliations:** 1 Department of Orthopaedics, 920th Hospital of the Joint Logistics Support Force of the PLA, 212 Daguan Road, Kunming, 650032 China; 2 Department of Radiology, 920th Hospital of the Joint Logistics Support Force of the PLA, 212 Daguan Road, Kunming, 650032 China

**Keywords:** Chronic osteomyelitis, Femur, bone defect, Internal fixation, Plate augmentation, Masquelet technique

## Abstract

**Background:**

To evaluate the effectiveness of a sequential internal fixation strategy and intramedullary nailing with plate augmentation (IMN/PA) for bone reconstruction in the management of infected femoral shaft defects using the Masquelet technique.

**Methods:**

We performed a retrospective descriptive cohort study of 21 patients (mean age, 36.4 years) with infected bone defects of the femoral shaft treated by the Masquelet technique with a minimum follow-up of 18 months after second stage. After aggressive debridement, temporary stabilisation (T1) was achieved by an antibiotic-loaded bone cement spacer and internal fixation with a bone cement–coated locking plate. At second stage (T2), the spacer and the locking plate were removed following re-debridement, and IMN/PA was used as definitive fixation together with bone grafting. We evaluated the following clinical outcomes: infection recurrence, bone union time, complications, and the affected limb’s knee joint function.

**Results:**

The median and quartiles of bone defect length was 7 (4.75–9.5) cm. Four patients required iterative debridement for infection recurrence after T1. The median of interval between T1 and T2 was 10 (9–19) weeks. At a median follow-up of 22 (20–27.5) months, none of the patients experienced recurrence of infection. Bone union was achieved at 7 (6–8.5) months in all patients, with one patient experiencing delayed union at the distal end of bone defect due to screws loosening. At the last follow-up, the median of flexion ROM of the knee joint was 120 (105–120.0)°.

**Conclusions:**

For infected femoral shaft bone defects treated by the Masquelet technique, sequential internal fixation and IMN/PA for the reconstruction can provide excellent mechanical stability, which is beneficial for early functional exercise and bone union, and does not increase the rate of infection recurrence.

## Background

A critical-size infected bone defect is a devastating complication of a fracture. Its management poses two main challenges to orthopaedic surgeons, namely, eradication of the infection and reconstruction of the large bone defect. While aggressive debridement is the most important option for eradiating infection, there are various kinds of treatment modalities for bone reconstruction, including Ilizarov’s bone transporting technique, vascularised bone transferring technique, and the Masquelet induced membrane technique [1–3].

During the last two decades, the Masquelet technique has evolved as a mainstay in the treatment of critical-size bone defects, considering that it has many advantages such as simple operation, relatively short time of bone union, and few complications [4, 5]. The technique consists of two stages: in the first stage (T1), after debridement, a polymethyl methacrylate (PMMA) bone cement spacer is used to fill the bone defect and induce a biological membrane formation; 6–8 weeks later, the spacer is removed and autologous bone graft is implanted for bone reconstruction in the second stage (T2) [6]. For an infected bone defect, combined with an aggressive debridement of the dead bone and soft tissue and the use of an antibiotic-loaded bone cement spacer, the Masquetlet technique has been proven efficient by our previous reports and other literature [7, 8].

The femur is a high-incidence site of chronic osteomyelitis and is affected in 24.46–26.8% of chronic osteomyelitis cases in southern China [9, 10]. When a large bone defect is formed following debridement, due to the coexistence of sagittal and coronal curvatures at the femur [11], there is discrepancy between mechanical and anatomical axes, resulting in high varus loads, which is less favourable for successful infection eradication and graft corticalisation. Therefore, in the treatment of infected femoral shaft bone defects with the Masquelet technique, selection of an appropriate and stable bone fixation method is essential, whether at T1 or T2. Although external fixation seems be the safest approach in such a situation, internal fixation should always be preferred for the femur because of the mechanical and functional reasons [12]. At T2, stable internal fixation is critical to achieve bone union. Furthermore, conversion from external to internal fixation at T2 raises the question of secondary infection related to pin tract contamination [13]. Thus, sequential internal fixation is a valuable option for controlling infection and bone reconstruction.

The optimal fixation method of the femur at T2 has been subject to debate, and practice varies widely from one surgeon to another depending on their habits. Intramedullary nail (IMN) fixation is the gold standard for the treatment of femoral shaft fractures. Although IMN is the treatment of choice with a good bone union rate, 1.1–14% of patients may still experience non-union [14, 15]. The main reason for bone non-union is that IMN has insufficient stability on femoral rotation and angulation [16]. Replacing the IMN with a new IMN with a larger diameter is a good choice for femoral shaft non-union after IMN fixation, but IMN with augmentation plate (IMN/AP) has shown a higher union rate and shorter operation time because it improves the biomechanical environment of the fracture site without causing more biological damage [17, 18].

To the best of our knowledge, there has been no clinical investigation of the sequential internal fixation and IMN/PA fixation stability at T2. The purpose of our retrospective descriptive cohort study was to evaluate the clinical efficacy of sequential internal fixation and IMN/AP for treating infected femoral shaft bone defects with the Masquelet technique. We hypothesised that sequential internal fixation and IMN/PA fixation at T2 can provide excellent mechanical stability for bone reconstruction in treating infected femoral shaft bone defects.

## Methods

### Inclusion and exclusion criteria

From January 2016 to January 2022, a total of 73 patients with infected femoral bone defect were treated with the Masquelet technique in our institution. The inclusion criteria for the study were as follows: a segmental bone defect of the femoral shaft after debridement with the length of the defect exceeding 4 cm; treatment by the two-stage Masquelet technique, including antibacterial-coated internal fixation at T1 and IMN/PA fixation at T2; physiological Cierny–Mader class A or B; age between 16 and 60 years; availability of regular radiographic and clinical follow-ups for more than 18 months after the operation at T2. The exclusion criteria were the incomplete follow-up data. This study was approved by our institutional review board.

After applying the inclusion and exclusion criteria, a total of 21 patients (18 male and 3 female) were included in this study. They had an average age of 36.4 years (age range, 17–60 years). Seventeen patients had post-traumatic osteomyelitis, while four patients had hematogenous osteomyelitis. Twelve patients had a sinus tract, and one patient had a pathological fracture at the time of admission. The average infection duration was 7.3 years (range, 0.3–44 years), and the mean number of operations performed before admission was 1.9 (range, 0–4 procedures). Five patients had knee joint ankylosis. Table 1 summarises the individual patients’ data. All the patients’ clinical and imaging results were retrospectively analysed.

**Table 1:** Patients’ characteristics

Case	Sex	Age (years)	Aetiology	Duration of infection (years)	Number of previous operations	Sinus tract	Knee joint ankylosis	Comorbidities
1	M	25	PTOM	12	1	N	N	N
2	M	30	PTOM	6	2	Y	Y	N
3	M	56	CHOM	50	1	N	N	Coronary disease
4	M	33	PTOM	3	0	Y	N	N
5	M	17	PTOM	2	3	N	N	N
6	M	21	CHOM	3	3	Y	N	N
7	M	26	CHOM	5	2	N	N	N
8	M	31	PTOM	1	2	N	N	N
9	M	59	PTOM	14	3	Y	Y	Post sigmoidostomy
10	M	50	PTOM	0.4	1	N	N	2DM
11	M	27	PTOM	2	4	N	N	N
12	F	27	PTOM	0.7	2	Y	N	N
13	F	44	PTOM	4	4	Y	N	N
14	M	22	PTOM	3	2	Y	N	N
15	M	37	PTOM	10	0	Y	Y	N
16	M	29	PTOM	0.6	4	Y	N	N
17	M	57	PTOM	1	2	N	N	2DM, gouty arthritis
18	M	60	PTOM	0.5	1	Y	N	N
19	F	53	PTOM	37	1	Y	Y	N
20	M	37	CHOM	3	1	Y	N	2DM
21	M	24	PTOM	0.3	0	N	Y	N

F female, M male, PTOM post-traumatic osteomyelitis, CHOM chronic haematogenous osteomyelitis, N none, Y yes, 2DM diabetes mellitus type 2

### Surgical procedure

The routine surgical protocol of the Masquelet technique was similar to that previously reported by our institution [19]. At T1 stage, aggressive debridement, fixation with a locking plate coated with Vancomycin (Eli Lilly, Tokyo, Japan)-loaded PMMA cement (Palacos^®^ R + G, Heraeus, Germany) and subsequent filling of the bone defect with cement was performed. At T2 stage, removal of the cement, re-debridement, replacement of fixation (locking plate + IMN), and placement of an autologous iliac bone/allogeneic bone graft were performed.

**Fig. 1:**
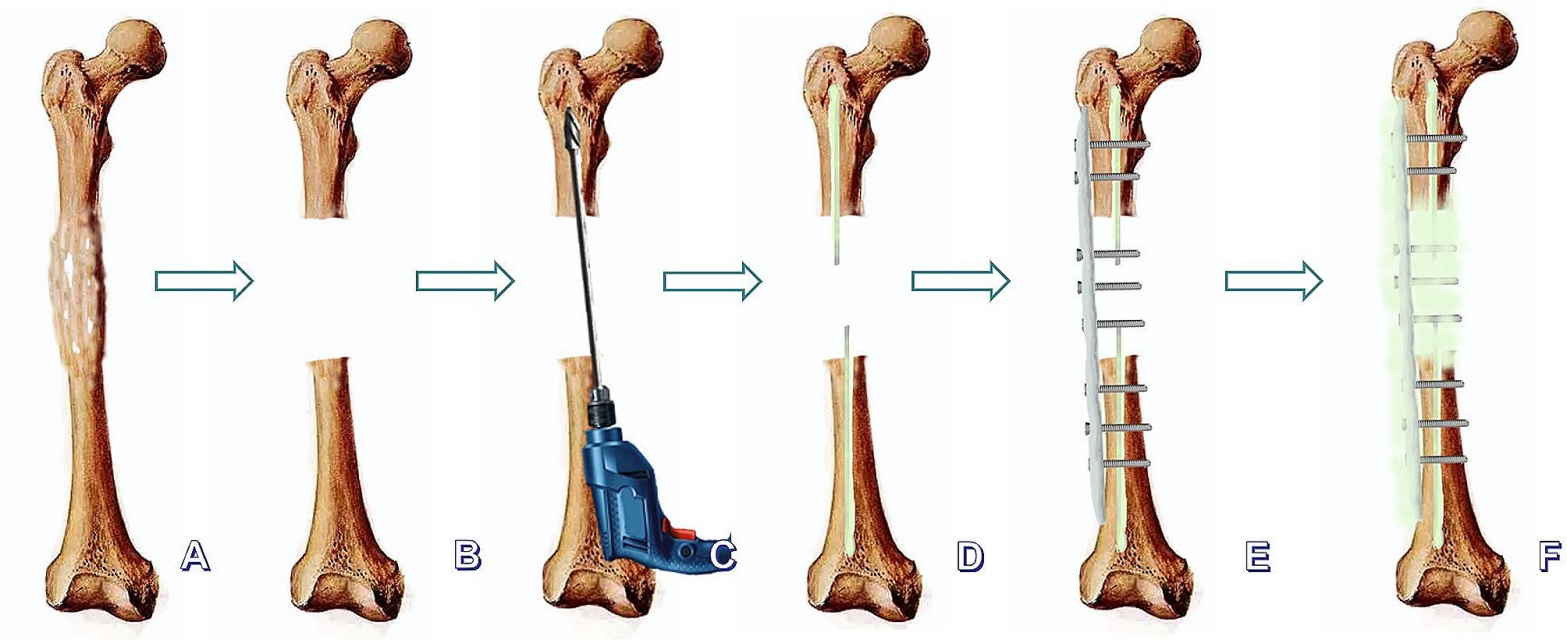
The flow chat of Chongqing technique. **A**: Chronic osteomyelitis of the femoral shaft (Cinery-Mader IV); **B**: The status after aggressive debridement by *en-bloc* resection of the lesion ; **C**: Debridement of combined with infection of the medullary cavity with electric drill and rigid reamer; **D**: The antibiotic-loaded cement rods were inserted into the medullary cavities of both bone ends; **E**: A locking-compression plate was used to fix both bone ends; **F**: The antibiotic-loaded cement was filled into the bone defect, coated on the surface of the plate and part of the bone ends. An antibiotic cement-plate composite device was formed

The treatment began with the debridement of all infected soft tissues and bone. First, the original internal fixation was removed. The range of debridement was determined by pre-operative magnetic resonance imaging (MRI), computed tomography (CT) scan, and radionuclide examination. The involved bone was resected segmentally (en bloc) until punctate bleeding appeared at the broken end (Paprika sign). In patients with concomitant intramedullary infection, repeated reaming with rigid reamers was also required to completely remove the infected tissue from the inner wall of the medullary cavity. At more than three sites of the bone defect area and the medullary cavity, at least three pieces of deep samples were harvested for microbiological analysis. At this point the assistant begins to configure the antibiotic bone cement, 2–4 g of Vancomycin was added into 40 g bone cement. After debridement and copious irrigation, two 1.2-mm Kirschner pins wrapped with antibiotic bone cement were made into cement rods with a diameter of about 7–10 mm, which were hardened and inserted into the distal and proximal medullary cavities from the bone defects, a locking plate was used to fix both bone ends. The length of plate depends on whether at least 2–3 screws on each side can be used to fix the both bone ends, and 2–4 screws were placed in the region of bone defect in order to combine the cement and plate into a collation. The alignment and length of the affected limb were checked by fluoroscopy. Then, the screw holes were filled with bone wax, and bone cement was wrapped to the plate and filled the bone defect, in line with the Chongqing technique(Fig. 1). Two to three drainage tubes were placed, and the surgical incision was closed layer by layer. Post-operatively, the patients were treated systematically with antibiotics based on the result of microbiological analysis of deep samples for at least six weeks. The affected limb was allowed to carry out exercise at the knee joint without weight-bearing as early as possible. White blood cells (WBC), C-reactive protein (CRP), and erythrocyte sedimentation rate (ESR) were measured every two weeks.

T2 operation was performed at least 8 weeks after the infection had been controlled. The wound of the T1 and the induced membrane were incised sharply. The bone cement spacer and the locking plate were removed. After re-debridement and rinsing by 3000 ml physiological saline, both ends of the resected bone margins were freshened, the medullary cavity was reamed, and an IMN was inserted and locked by screws. The alignment and length of the affected limb and the location of the IMN were checked by fluoroscopy. Then, a narrow reconstructive locking plate was placed on the lateral or anterior cortex of the femoral bone defect, the screws penetrated one cortex, and at least 2–3 screws were fixed at each end to further fix the bone ends. Subsequently, cancellous bone harvested from the posterior superior iliac spine was cut into 2mm^3^ pieces and implanted into the bone defect. If the autograft bone was insufficient, allogeneic bone could be mixed, but the ratio of allogeneic bone had to be less than 25%. Antibiotics were continued for another two weeks. Post-operatively, no weight-bearing was allowed on the affected limb within three months. Meanwhile, exercise of the adjacent joints was started after the wound had healed. After three months, weight-bearing was gradually allowed depending on the result of radiographic examination.

### Follow-up

After T2 operation, the patients were instructed to perform the routine follow-up visits at 1, 2, 3, 6, 9, and 12 months, and then every 3–6 months. The content of the follow-up visits included radiography of the affected limbs; WBC, ESR, and CRP; and other clinical presentations (such as local redness and swelling, purulent secretion, or sinus tract). Radiographs were taken to evaluate the state of bone union. Radiographic bone union was defined as the presence of bridging callus between the bone graft and both bone ends (bone bridge in at least three out of four cortices) on the anteroposterior (AP) and lateral radiographs. Non-union was defined as there is a gap between the bone end and the bone graft at 6 months after T2 surgery and still existence at the following 3 months on radiographs. WBC, ESR, CRP, and other clinical presentations were assessed to determine whether there was recurrence of infection. The recurrence of infection was defined as local redness and swelling, purulent secretion or sinus tract, and with/without the increasing of WBCs, CRP, and ESR.

The data of the bone defect’s length after debridement, the interval time between T1 and T2, the time of follow-up, radiographic bone union time and full weight-bearing time, the range of motion (ROM) of the affected limb’s knee joint, and complications (including infection recurrence, donor site pain, delayed wound healing, discrepancy, refracture, and ankylosis) were collected. A result was considered “good” when the patient had no recurrent infections, radiological imaging showed bone union, and he or she was able to walk with full weight-bearing at the last follow-up.

### Data analysis

SPSS 25.0 (IBM Corp., Armonk, NY, USA) was used for statistical analysis. The Shapiro-Wilk test was used to test whether data had a normal distribution. The continuous data were expressed as means ± standard deviation. Data with non-normal distribution were expressed as the median (interquartile range). Categorical data were summarized using ratios and percentages.

## Results

The operations of all 21 patients were performed by the same surgeon (J. S.), and were completed in line with the pre-operative plan. After debridement, the median and quartiles of bone defect length was 7 (4.75–9.5) cm. After T1, four patients required iterative debridement with induced membrane excision and spacer and internal fixation exchange due to infection recurrence. Two patients were relieved after one re-debridement, while the other two achieved infection control after re-debridements two and four times. Eleven patients had positive bacterial cultures. The causative pathogen was Staphylococcus aureus in seven patients, Escherichia coli in two cases, and Pseudomonas aeruginosa in two cases.

The median T1–T2 interval was 10 weeks (9–19 weeks). The volume of the bone defect was estimated based on CT measurement before T2 surgery. The mean volume of the bone defect was 79.74 ± 31.52 cm^3^. The median follow-up period after T2 surgery was 22 months (20–27.5 months). The median radiographic bone union occurred in 7 months (6–8.5 months). Twenty patients achieved bone healing within 11 months, and one patient achieved bone healing only after 16 months due to the loosening of the screws of the augmentation plate. Full weight-bearing was noted at 10.48 ± 2.84 months. In T2 stage, there was no infection recurrence at the last follow-up. Three patients showed delayed healing of the wounds at the posterior superior iliac spine bone-harvesting sites. After suture removal, rinsing, and repeated dressing changes, two patients’ wounds healed spontaneously, and one patient’s wound healed after re-debridement and re-suturing. In two cases, pain at the bone donor site that remained within three months after operation was gradually relieved within six months after operation. Bone resorption under the plate occurred in two patients after operation. The augmentation plates were removed, and local bone re-grafting was performed. Table 2 summarises the management procedures and results in each of the patients.

**Table 2:** Patients’ clinical data at the last follow-up

Case	Length of bone defect (cm)	Re-debridement	Pathogenic bacteria	Interval between T1 and T2 (weeks)	Volume of bone defect (cm^3^)	Follow-up time (months)	Radiological bone union (months)	Full weight-bearing (months)	Flexion ROM of knee joint (°)	Complications
1	5	N	*S. aureus*	23	57.2	19	6	13	120	N
2	12	N	*E. coli*	15	125.6	22	7	10	45	DHDS
3	8	N	(-)	38	81.3	52	7	11	120	N
4	4.5	N	*S. aureus*	19	50.3	21	7	10	120	PDS
5	7	N	(-)	9	71.0	18	5	6	120	N
6	7	N	(-)	9	73.4	23	7	10	120	N
7	11	Y	*P. aeruginosa*	11	100.8	22	10	15	120	DHDS
8	16	N	(-)	8	164.6	39	8	10	90	Bone resorption partly
9	8	N	*S. aureus*	11	98.7	32	16	16	10	Screws loosing & delayed bone union
10	5.5	N	*P. aeruginosa*	9	61.2	24	8	8	120	N
11	4	N	*S. aureus*	58	40.6	18	7	11	120	N
12	9	N	(-)	9	83.3	43	8	9	120	DHDS & PDS
13	4.5	Y	*S. aureus*	10	49.8	27	6	7	120	N
14	4	N	(-)	13	52.6	20	7	9	120	N
15	5.5	N	(-)	36	58.8	24	6	6	30	N
16	8	N	(-)	8	84.1	22	9	11	120	N
17	7	N	*S. aureus*	9	87.2	20	8	10	120	N
18	4.5	Y	*E. coli*	9	44.8	28	12	16	120	N
19	10	Y	*S. aureus*	10	98.7	21	6	9	30	Bone resorption partly
20	5	N	(-)	19	62.4	19	9	10	120	N
21	12	N	(-)	9	128.2	22	6	13	120	N

Y: yes, N: none, E. coli: Escherichia coli, S. aureus: Staphylococcus aureus, P. aeruginosa: Pseudomonas aeruginosa, ROM: range of motion, DHDS: delayed healing of donor sites, PDS: pain at the donor site

**Fig. 2:**
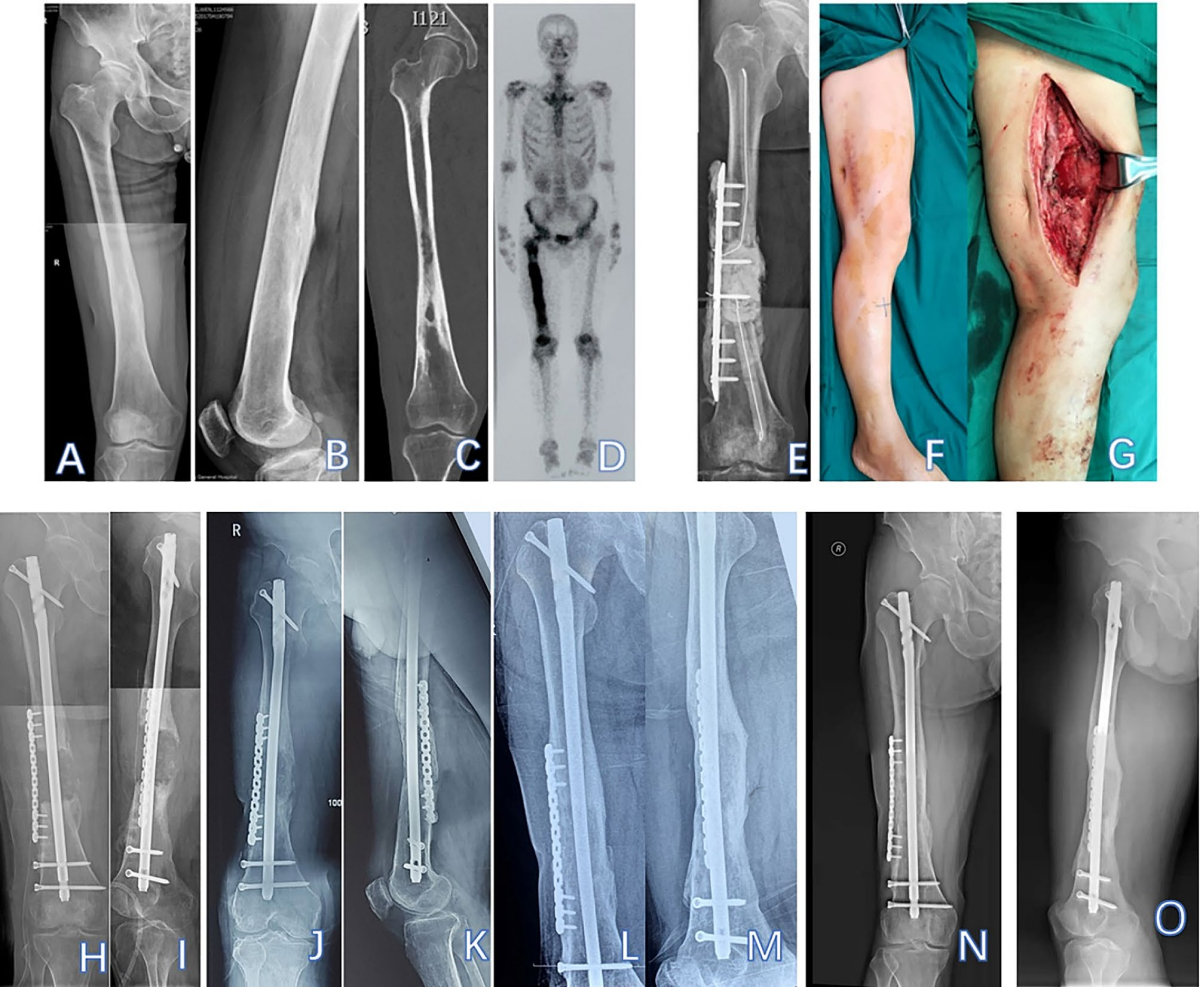
Patient #3, a 56-year-old man with Cinery–Mader type IV chronic haematogenous osteomyelitis in the shaft of the right femur. **A**: Pre-operative anteroposterior (AP) radiograph of the right femur; **B**: Pre-operative lateral X-ray of the right femur; **C**: Pre-operative CT shows bone destruction; **D**: Pre-operative bone radionuclide scintigraphy; **E**: Radiograph shows that the lesion was segmentally resected, the bone defect was filled with antibiotic-loaded bone cement, and a cement-coated locking plate was used as temporary stabilisation at T1; **F**: Wound healing before T2 surgery; **G**: After the bone cement was removed, an intact induced membrane could be observed. **H & I**: Radiograph shows an IMN and a locking plate that were used as definitive fixation after T2 surgery; **J & K**: Callus growth was observed after 5 months; **L & M**: AP and lateral radiographs show bone union after 30 months; **N & O**: Radiographs after 52 months

At the last follow-up, the median flexion ROM of the knee was 120°(90°-120.0°). The hip range of movement was normal in all of the patients, and all patient treatment outcomes were considered good (Fig. 2).

## Discussion

The most common complications of the Masquelet technique for treating infected bone defects include infection recurrence and non-union of the bone defect [13]. The insufficiency of mechanical stability is one of important causes of non-union in the definitive bone fixation at T2 stage [13]. The insufficient stability can affect the biological process of graft revascularisation by rupture of the inner membrane vascular buds, leading to non-union and subsequent implant failure [20]. The results of the present study showed that sequential internal fixation and IMN/PA for the treatment of infected bone defects of the femoral shaft with the Masquelet technique resulted in good clinical outcomes in all patients. Despite the different causes of bone infection, none of the patients experienced the recurrence of infection, and the radiographic union rate of the bone defect was achieved in 94.7%, considering that only one patient had delayed union at the last follow-up due to the loosening of the screws of the plate.

The method of stability after debridement of infected bone defects in the T1 stage includes external and internal fixation. The choice depends on the site of the defect [19]. For femoral shaft defects, both options are available. External fixation can reduce the risk of additional infection associated with implants by spanning the infected region. However, the use of an external fixator often causes many negative effects, such as difficult postoperative care, high incidence of pin loosing and pin-tract infection, joint stiffness, etc [19]. In recent years, internal fixation has become a preferred method of stability, especially in the femur and upper extremity [13, 21]. Wang et al [9], in a retrospective analysis of 356 patients, found that induced membrane technique for repair of bone defects should be preferred to internal fixation because it reduces complications and does not increase recurrence of infection. Our previous study compared the effect of external fixation with internal fixation on infection recurrence and bone healing after debridement of post-traumatic tibial osteomyelitis and found that the use of internal fixation did not increase the rate of infection recurrence or affect bone union [19]. Therefore, internal fixation is a good choice.

The choice of internal fixation method at T1 is now controversial. Antibiotic cement-coated IMN provides strong stability in long bone defects and allows early weight bearing [22]. However, previous studies and the Meta-analyses have shown that the use of IMN in T1 stage increases the rate of infection recurrence after T2 [23, 24]. In contrast to IMN, plates ensure precise correction of the axes and length of the limb and provide stable static fixation, allowing early exercise of the affected limb. However, the surface of the plate is prone to biofilm formation, leading to recurrence of infection. It has previously been reported that the use of bone cement-coated plate composite device following debridement at T1 is a reliable treatment method (“Chongqing technique”) [25]. Yu et al. [26]. used locking plate covered with antibiotic bone cement as internal fixation to treat femoral chronic osteomyelitis at T1. In addition to significantly improving patient comfort and increasing stability, it does not increase the rate of recurrence of infection. In our experience, this composite offers at least three advantages compared with other methods. First, it can deliver high concentrations of antibiotics to the bone defect over a long period of time. This provides benefits in killing residual planktonic bacteria, thereby preventing biofilm formation and reducing the recurrence of infection. Secondly, it integrates the plate and the bone cement spacer into a whole, thereby forming a “reinforced concrete structure” for promoting some degree of bone stability. Furthermore, this ensures to form an intact induced membrane. Third, it expands the volume of the spacer, because the spacer should be slightly larger than the diameter of the bone, which can avoid smaller bone diameters at the site of the bone defect due to partial resorption of the grafted bone [4].

In this study, the high rate (19.04%, 4/21) of infection recurrence after initial debridement of T1 remained the main factor of prolonged treatment cycle. Although local stability and filling the antibiotic-loaded PMMA bone cement into bone defect can reduce the recurrence rate, previous studies have revealed that insufficient debridement is the main cause [27]. This is consistent with our view. The abundant soft tissues arounding the femur makes it difficult to expose, resulting in unclear display of lesions during debridement, leading to insufficient debridement, especially some smaller abscesses located in muscle tissues are often easy to be ignored. Therefore, preoperative MRI of the affected limb is particularly important to understand in detail the number, location and extent of lesions in the soft tissue, which has a positive effect on reducing the recurrence rate of infection.

At T2, the controversy over stability methods for bone reconstruction of the femoral shaft defects continues. IMN can provide optimal restoration of the mechanical axis in large long-bone defects, rigid stability, and good tolerance in the long term. Morwood et al. [28]. demonstrated the superiority of IMN over plates in the application of the Masquelet technique to the femur. In their view, IMN has many advantages, such as faster union, less bone graft, and fewer reoperations for any cause compared with those treated with plates. They attributed this result to both the development of an intramedullary canal and the circumferential stress on the graft with early weight-bearing when using an IMN, as opposed to a certain degree of stress shielding and delayed weight-bearing when using plate fixation. Giannoudis et al. [29]. conducted a prospective study to evaluate the results of treatment of bone defects with the Masquelet technique in 43 patients, including 10 patients with femoral bone defects that were fixed with IMN. During the follow-up, two patients experienced bone non-union in one of the defect sides requiring re-grafting. Unfortunately, both of them were the patients with the femoral bone defect fixed with IMN. Yang et al. [30]. suggested that the stability of IMN is mainly achieved by the tight contact with the intramedullary cavity, and the lack of the tight contact aggravates rotational instability, which is the main mechanical problem for the failure of IMN fixation in the femur.

In the critical-size bone defects of the femoral shaft, the extensive loss of the medullary cavity results in a significant reduction in the contact area between the IMN and the inner surface of the medullary cavity, leading to further rotational instability, which may be insufficient for bone graft integration within the Masquelet technique [31]. In such situations, IMN can be combined with an additional fixation method [13]. Walcher et al. [32]. compared augmentative plating using a locking compression plate leaving the IMN with the exchanging of IMN in a saw-bone model of a femoral shaft non-union. Their results showed that the difference of fracture gap motion in axial testing was small, but the IMN allowed for the largest amount of motion in rotational testing. The augmentative plate was the most stable construct in all loading conditions. They concluded that the augmentative plating while leaving the nail in situ is biomechanically superior to exchanging IMN. Park et al. [33]. compared the differences in mechanical rigidity between PA and IMN using a cadaveric fracture model of the femur. They found a 2.6-fold increase in bending stiffness and a 3.3-fold increase in torsional stiffness in plate augmentation compared with IMN. This fixation option allows advantages of IMN to be maintained while increasing stability. In our study, we found micromovement between the ends of the bone defect after fixation with IMN, which is a risk factor for bone non-union in future. By placing the augmentation plate on the lateral or anterior cortex of the femoral shaft, this micromovement can be eliminated.

The augmentation plate is one of the main treating methods for the femoral shaft non-union after IMN fixation, because it can achieve axial, anti-rotation, and anti-angulation mechanical stability [34]. Perisano et al. [15]reviewed 24 studies comprising a total of 502 patients treated with augmentation plate in the femoral shaft non-union after IMN. A complete bone union was achieved in 5.8 ± 2.1 months in 98% of the patients. The results showed that augmentation plate has a high rate of consolidation in the femoral shaft non-union, with good functional recovery and a low incidence of complications. In our study, 21 patients with infected femoral bone defects were included, with a mean bone defect length of 7.5 cm; 20 of them achieved bone union after not more than 12 months, indicating the reliability of this approach. The results showed that rigid fixation facilitated early functional exercise of the affected limb, which could prevent ankylosis of the knee joint. One patient presented screw loosening of the plate that occurred 1 month after T2 surgery, which resulted in delayed union of the distal end of the bone defect until 15 months later. Throughout the whole process, due to the patient’s refusal, we did not perform re-fixation and re-grafting, and only requested that the affected limb be prohibited from weight-bearing, and eventually obtained bone union. It suggests, in part, that the delayed union in this patient may have been caused by mechanical instability due to the lack of plate assistance.

Double plate fixation can also provide adequate mechanical stability in three planes to counter shear effect and has been considered the preferred option for treating long-bone fracture non-union [35]. Maimaitiyiming et al. [36] retrospectively analysed 14 patients with the femoral shaft non-union after IMN, and their patients underwent double plate fixation and bone grafting. The mean follow-up was 14.8 months (range, 10–25 months). All of the patients achieved union, on average, in 5 months and 2 weeks (range, 4–7 months). None of the patients had internal fixator loosening or breakage, deformity, and infection. However, Zhang et al. [34]. compared clinical outcomes between double plating and exchange nailing with augmentation plating in 30 patients with the femoral shaft non-union. Compared with double plating, augmentation plating resulted in a shorter incision, faster fracture healing, and shorter time to return to work. Furthermore, the application of IMN also means a reduction in the amount of bone graft, which is of great significance for the critical-size femoral shaft defects.

Although the method of PA/IMN is regarded as a promising treatment for the femoral shaft non-union, the way in which the plate is fixed remains controversial. Due to the anterior femoral curvature and obstruction from the IMN, achieving bicortical screw fixation is very difficult [37]. In our study, all of the patients underwent unicortical screw fixation using a locking plate. This was because the method is simple and efficient. The surgical time was shortened, and 94% of the patients did not experience screw loosening and achieved bone union within 8.4 months. Furthermore, Nadkarni et al. [38]. showed a good result of this procedure in the management of the femoral shaft non-union. They suggested that the locking plates by virtue of their angular stability could give a superior hold over the conventional compression plates. However, unicortical fixation with locking screws still has a tendency to loosen screws, resulting in fixation failure. Therefore, when bicortical fixation with screws is possible, it is recommended. Wu and his colleagues [37] designed a multidimensional cross locking plate. It provides multidimensional stability via bicortical screw fixation, where the locking screws are drilled at a 30-degree divergent angle in the coronal plane to clamp the IMN, thereby obtaining a faster bone healing rate and satisfactory functional recovery.

There are some limitations to this study. It was a retrospective descriptive and a single-centre study, and with this, the ability to fully evaluate the method was limited. Furthermore, the sample size was small, and there was no other method to compare the results with. Another limitation is that the mean follow-up period was relatively short, and longer-term follow-up is required to observe the effect on infection control and bone reconstruction.

## Conclusion

For treating infected femoral shaft bone defects, sequential internal fixation and IMN/PA fixation at T2 can provide excellent mechanical stability. It seems to be a valuable option for the reconstruction of infected femoral shaft bone defects by the Masquelet technique, which is beneficial for early functional exercise and bone union.

## Data Availability

Data is provided within the manuscript or supplementary information files.

## Abbreviations

IMN/PA: Intramedullary nailing with plate augmentation
PMMA: Polymethyl methacrylate
IMN: Intramedullary nail
MRI: Magnetic resonance imaging
CT: Computed tomography
WBC: White blood cells
CRP: C-reactive protein
ESR: Erythrocyte sedimentation rate
AP: Anteroposterior
